# MAISNet: a multi-species integrated graph neural network for acetylcholinesterase inhibitor screening

**DOI:** 10.1093/bioinformatics/btag153

**Published:** 2026-04-15

**Authors:** Dan Shao, Shengjie Liang, Yucong Xiong, Guangmin Liang

**Affiliations:** Department of Artificial Intelligence, College of Computer Science and Technology, Changchun University, Changchun 130022, China; Department of Artificial Intelligence, College of Computer Science and Technology, Changchun University, Changchun 130022, China; College of Computer Science and Technology, Jilin University, Changchun 130000, China; Information Security and Management Undergraduate School of Artificial Intelligence, Shenzhen Polytechnic University, Nanshan District, Shenzhen, Guangdong Province, 518000, China

## Abstract

**Motivation:**

Global population aging has led to a rapid increase in neurodegenerative disorders such as Alzheimer’s disease (AD). Although existing drugs can temporarily alleviate symptoms, none have been proven to delay or prevent disease progression. Acetylcholinesterase inhibitors (AChEIs) have been shown to mitigate AD symptoms, yet traditional AChEI screening approaches remain time-consuming and inefficient.

**Results:**

To address this limitation, we developed multi-species AChEI screening network (MAISNet), an AChEI screening framework based on acetylcholinesterase (AChE) data from six species. In MAISNet, inhibitor molecules were represented as SMILES-derived molecular graphs, whereas AChE protein structures were encoded as residue contact maps. Multi-scale molecular and protein features were extracted using the sample and aggregate (GraphSAGE) network and the graph attention network, respectively, and were subsequently fused through a bidirectional cross-attention mechanism. The integrated representations were then processed by a multilayer perceptron (MLP) to inhibitor classification. On both internal and external validation sets, MAISNet consistently outperformed five baseline models. Furthermore, we applied MAISNet to screen existing small molecules, and Methyl 2-[(3S)-3–(1, 2, 3, 4, 5, 6, 7, 8-octahydro-2-naphthyl)-2-(methoxycarbonyl)-1H-pyrrol-1-yl]acetate subsequently emerged as the top-ranked candidate. Overall, MAISNet significantly improves the accuracy and generalization capability of AChEI screening, providing an efficient and reliable computational tool for accelerating therapeutic discovery for AD.

**Availability and implementation:**

Code that supports the reported results can be found at: https://github.com/liangshengjie111/MAISNet. The archival version of the code is preserved on Zenodo at https://doi.org/10.5281/zenodo.18721665.

## 1 Introduction

With the acceleration of global aging, neurodegenerative diseases such as Alzheimer’s disease (AD) have become pressing public health concerns (Zhang *et al*. 2024). A growing body of evidence indicates that disturbances in acetylcholine (ACh) signaling are closely linked to the progression of AD, particularly within the basal forebrain cholinergic system (Hampel *et al.* 2018). In AD, cholinergic neurons in the basal forebrain—the primary source of ACh innervation to the hippocampus, amygdala, and cortical mantle—undergo progressive degeneration, leading to a substantial reduction in ACh levels in these brain regions (Whitehouse *et al*. 1982). Acetylcholinesterase (AChE), the enzyme responsible for degrading ACh in the synaptic cleft, exhibits elevated activity in AD, thereby accelerating ACh breakdown and further exacerbating cholinergic deficiency (Chen *et al*. 2022). To counteract this process, acetylcholinesterase inhibitor (AChEIs) specifically block AChE activity, slowing ACh degradation, enhancing synaptic ACh availability, and ultimately improving cognitive function (Galimberti and Scarpini 2016).

Extensive efforts have focused on developing AChEIs as therapeutic agents for neurodegenerative diseases. Among them, donepezil has been shown to improve both cognitive function and activities of daily living in patients with mild to moderate AD (Birks and Harvey 2018). Rivastigmine has demonstrated clinical benefits in AD as well as Parkinson’s disease dementia, with significant improvements in cognitive assessment scores (Meng and D’Arcy 2013). Galantamine, in addition to inhibiting AChE, also modulates nicotinic receptors, thereby exerting dual effects on cognitive enhancement (Lilienfeld 2002). While these drugs provide short-term symptomatic relief, none have been proven to slow or halt the long-term progression of AD (Salloway *et al*. 2019). Consequently, the development of novel therapeutic strategies remains an urgent necessity.

Traditional drug discovery methods, such as high-throughput screening, are heavily reliant on extensive experimental procedures, rendering them both time-consuming and costly. Furthermore, the intricate relationship between molecular structure and biological activity often limits the effectiveness of these conventional approaches, which struggle to capture the full complexity of molecular interactions. In recent years, artificial intelligence techniques have emerged as powerful tools in the study and development of AChEIs, and have also achieved remarkable performance in other biomolecular prediction tasks (Shao *et al*. 2022). For instance, Zhu *et al*. employed graph neural networks (GNNs) to encode molecular graph representations and predict potential inhibitory compounds (Wieder *et al*. 2020). Liu *et al*. developed a deep convolutional neural network-based model to predict drug-target interactions, aiming to estimate the inhibitor classification between AChE and candidate molecules (Lee *et al*. 2019). Mendes *et al*. utilized random forest algorithms to predict the inhibitory activity of various molecules against AChE (Niu *et al*. 2017). Additionally, Biedenkapp *et al*. integrated bioinformatics with ML techniques to streamline and optimize the AChEI screening process (Khan *et al*. 2022). Despite these advances, current computational models remain limited in their ability to fully capture the complex, multilevel interactions between proteins and ligands. Moreover, most studies have focused exclusively on human AChE, which constrains the identification of conserved mechanisms across different species.

**Figure 1:**
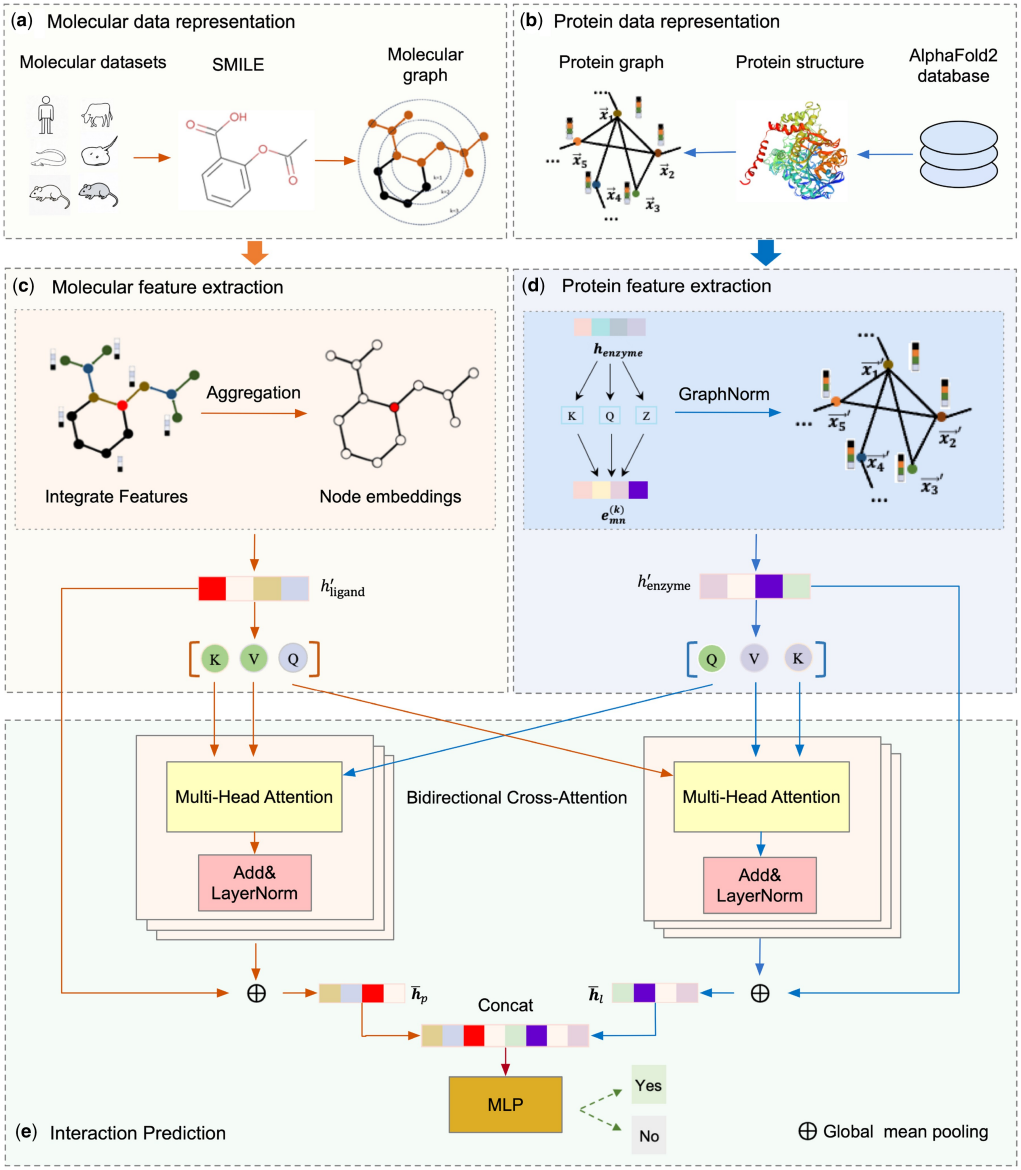
Overview of the proposed MAISNet framework. (a) Inhibitor molecules from six species—*Homo sapiens(human)*, *Electrophorus electricus(electric eel)*, *Mus musculus(house mouse)*, *Bos taurus(bovine)*, *Torpedo californica(Pacific electric ray)*, and *Rattus norvegicus(Norwegian rat)*—are represented as graphs derived from SMILES strings. (b) AChE protein structures obtained from the AlphaFold2 database are represented as residue contact map–based graphs. (c) Molecular features are extracted using a hybrid GNN encoder. (d) Protein features are extracted using a specialized GNN encoder. (e) A bidirectional cross-attention mechanism fuses molecular and protein representations, followed by a MLP to predict inhibitor classification and interaction strength for candidate screening.

To address the aforementioned challenges, we proposed a prediction method for AChEI interactions, termed the multi-species AChEI screening network (MAISNet), covering *Homo sapiens (human)*, *Electrophorus electricus (electric eel)*, *Mus musculus(house mouse)*, *Bos taurus(bovine)*, *Torpedo californica(Pacific electric ray)*, and *Rattus norvegicus (Norwegian rat)*, as illustrated in Fig. 1. In this framework, inhibitor molecules and AChE proteins were initially represented using SMILES string and residue contact maps, respectively. These graph-based representations were processed by two hybrid GNN encoders to extract structural and contextual features. A bidirectional cross-attention mechanism was then employed to enable multi-level interactions fusion between the inhibitor and AChE representations. A MLP was utilized to evaluate their inhibitor classification and interaction strength. Finally, all candidate molecules were evaluated using MAISNet based on their predicted binding affinities, and Methyl 2-[(3S)-3–(1, 2, 3, 4, 5, 6, 7, 8-octahydro-2-naphthyl)-2-(methoxycarbonyl)-1H-pyrrol-1-yl]acetate was selected as the top-ranked drug candidate.

Our key contributions are as follows:

- The first multi-species predictive framework for AChEI interactions, integrating data from six phylogenetically distinct species to improve accuracy and generalizability.
- Hierarchical dilated graph convolution captured multi-scale molecular features from local bonds to global scaffolds, with multi-head attention highlighting pharmacologically relevant atoms.
- Heterogeneous graph fusion architecture integrated protein contact maps and ligand graphs via bidirectional cross-attention, enhancing protein-ligand interaction modeling.

## 2 Materials and methods

### 2.1 Datasets

In this study, inhibitor molecule data were retrieved from the BindingDB database (https://www.bindingdb.org, accessed on 3 March 2024) (Chen *et al*. 2001). BindingDB is a specialized database focusing on protein-small molecule interactions, providing extensive data on inhibitor classification, including $K_{d}$, $K_{i}$, and ${\text{IC}}_{50}$, with over 2 million binding entries across thousands of proteins and drug-like molecules.

We selected AChE inhibitors from six species in the BindingDB database—*Homo sapiens*, *Electrophorus electricus*, *Mus musculus*, *Bos taurus*, *Torpedo californica*, and *Rattus norvegicus*—as our experimental dataset (Dataset A). We used the ${\text{IC}}_{50}$ of inhibitors as the criterion to classify positive and negative samples. Following the approach of Li *et al*. (2023b), positive samples were labeled as inhibitors with ${\text{IC}}_{50}\leq 1\;\mu M$. To balance the dataset, we randomly selected an equal number of negative samples from compounds with ${\text{IC}}_{50}>1\;\mu M$ (Guo *et al*. 2017).

To ensure the quality of the dataset and the biological relevance of the potential leads, a multi-stage medicinal chemistry filtering pipeline was implemented using the RDKit library. Beyond the removal of inorganic compounds and SMILES normalization, we applied rigorous drug-likeness filters to the initial pool. Specifically, molecules were required to adhere to Lipinski’s Rule of Five (molecular weight ≤ 500 Da, log P≤5, H-bond donors ≤ 5, and H-bond acceptors ≤ 10) to ensure favorable oral bioavailability (Lipinski *et al*. 2001). We further applied Veber’s criteria, restricting the number of rotatable bonds to ≤ 10 and the polar surface area to ≤ 140 ${Å}^{2}$, as these properties are highly correlated with good membrane permeability (Veber *et al*. 2002). Crucially, to eliminate “frequent hitters” and false positives in enzymatic assays, we performed a pan-assay interference compounds filtration to exclude molecules with chemically reactive or unstable substructures (Baell and Holloway 2010). These stringent filters ensured that the dataset consisted of molecules with genuine pharmacological potential, reducing noise and improving the overall quality of the data.

Ultimately, Dataset A consists of a total of 17 352 inhibitor molecules, comprising 6328 compounds from *Homo sapiens*, 7468 compounds from *Electrophorus electricus*, 728 compounds from *Mus musculus*, 668 compounds from *Bos taurus*, 304 compounds from *Torpedo californica*, and 1856 compounds from *Rattus norvegicus*. Of these, 8762 compounds are labeled as positive samples and 8590 compounds are labeled as negative samples. The dataset was then split into training, validation, and testing sets, with an 8:1:1 ratio, as shown in Table 1.

**Table 1:** Species composition and sample distribution in Dataset A and B.

	Dataset A							Dataset B		
Species	Total	Training		Validation		Testing		Total	Pos	Neg
		Pos	Neg	Pos	Neg	Pos	Neg			
Homo sapiens	6328	2538	2525	313	305	312	335	1450	812	638
Electrophorus electricus	7468	3008	2977	366	390	359	368	1449	703	746
Mus musculus	728	296	301	31	29	36	35	596	313	283
Bos taurus	668	255	263	43	36	36	35	523	305	218
Torpedo californica	304	115	117	17	18	20	17	371	199	172
Rattus norvegicus	1856	837	672	90	84	90	83	1449	743	706
Total	17352	7049	6855	860	862	853	873	5838	3075	2763

Abbreviations: Neg, negative; Pos, positive.

In addition, to evaluate model robustness, an external validation set (Dataset B) was curated from the ChEMBL database (https://www.ebi.ac.uk/chembl/, accessed on 7 August 2025) (Gaulton *et al*. 2011). Negative samples were generated using the same construction strategy as Dataset A. During the construction of Dataset B, compounds overlapping with the training set (Dataset A) were removed through SMILES-based deduplication. Additionally, the same molecular filtering strategy applied to Dataset A was also used for Dataset B. Ultimately, Dataset B comprised 5838 inhibitor molecules, including 3075 positive and 2763 negative samples, spanning six species: *Homo sapiens* (1450 compounds), *Electrophorus electricus* (1449), *Mus musculus* (596), *Bos taurus* (523), *Torpedo californica* (371), and *Rattus norvegicus* (1449).

### 2.2 Graph-based data representation

#### 2.2.1 Molecular data representation

To represent inhibitor molecules, we employed SMILES strings to encode their chemical structures. In the graph representation, atoms were treated as nodes, and chemical bonds as edges.

Each atom node $h_{\text{ligand}}\in R^{105}$ was encoded as a 105-dimensional feature vector comprising the following components:

1. a 100-dimensional one-hot encoding representing both common and rare atomic elements;
2. a 1-dimensional normalized formal charge;
3. a 1-dimensional normalized van der Waals radius;
4. a 1-dimensional binary indicator of aromaticity;
5. a 2-dimensional chirality descriptor.

The complete feature representation is given by:


(1) $$\begin{array}{cc} h_{\text{ligand}}=[ & \delta_{t_{1}},\delta_{t_{2}},\ldots ,\delta_{t_{100}},\frac{q_{j}-\mu_{q}}{\sigma_{q}},\\ & \frac{r_{j}^{\text{vdw}}-r_{\min}}{r_{\max}-r_{\min}},I_{\;\text{aromatic}},c_{R},c_{S}] \end{array}$$

where $\delta_{t_{i}}(i\in \{1,2,\ldots ,100\})$ denotes the one-hot encoding of atomic types; $q_{j}$ is the partial charge of the *j*-th atom; $\mu_{q}$ and $\sigma_{q}$ represent the mean and standard deviation of atomic charges, respectively; $r_{j}^{\text{vdw}}$ is the van der Waals radius of the *j*-th atom; $r_{\min}$ and $r_{\max}$ denote the minimum and maximum van der Waals radii across the dataset; $I_{\text{aromatic}}$ is a binary indicator of whether the atom is aromatic; and $c_{R}$ and $c_{S}$ represent the chirality descriptors.

#### 2.2.2 Protein data representation

In this study, AChE proteins were represented as graphs based on their structural information. Considering the limited coverage of experimentally resolved protein structures in the protein data bank (PDB), we utilized predicted structures provided by AlphaFold2, which has demonstrated outstanding performance in a wide range of protein function prediction tasks (Shao *et al*. 2025). Specifically, we obtained the structural data for six distinct AChE variants from the AlphaFold2 protein structure database (https://alphafold.com, accessed on 28 June 2025) (Jumper *et al*. 2021).

**Figure 2:**
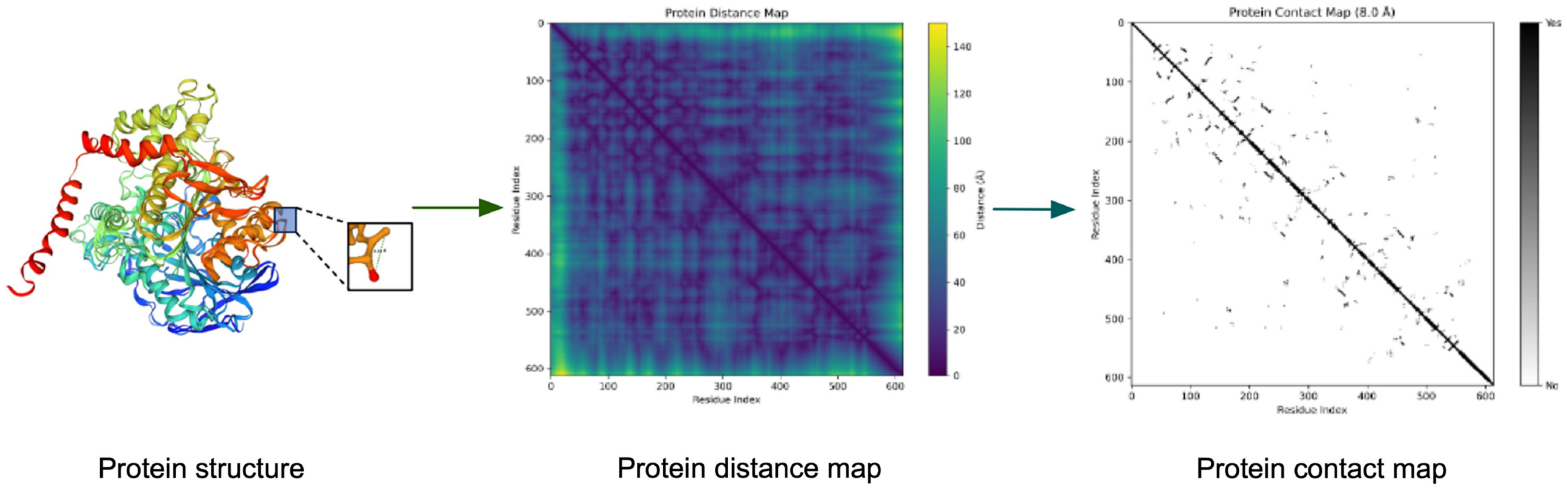
Protein graph representation. The three-dimensional structure of the AChE protein was used to compute pairwise residue distances, which were first converted into a distance map and subsequently transformed into a binary contact map.

Based on the coordinate information provided by AlphaFold2, each protein structure was transformed into a residue-level contact map derived from its 3D coordinates. This map was constructed by computing the Euclidean distances between all pairs of amino acid residues. An edge was added between two residues if the distance between them was less than 8.0 Å, thereby generating a graph-based representation of the protein structure, as illustrated in Fig. 2.

Each residue node $h_{\text{enzyme}}\in R^{24}$ was represented by a 24-dimensional feature vector comprising the following components:

1. a 20-dimensional one-hot encoding of the amino acid type;
2. a 3-dimensional secondary structure probability distribution—α-helix, β-sheet, and random coil—derived from the dictionary of protein secondary structure (DSSP) algorithm;
3. a 1-dimensional binary indicator of solvent accessibility, determined using a relative accessible surface area threshold of 25%.

The specific feature representation is defined as follows:


(2) $$h_{\text{enzyme}}=[\delta_{a_{1}},\delta_{a_{2}},\ldots ,\delta a_{20},P_{\alpha },P_{\beta },P_{\text{coil}},I_{\text{ASA}}]$$

where $\delta_{a_{i}}(i\in \{1,2,\ldots ,20\})$ denotes the one-hot encoding of the amino acid type; $P_{\alpha }$, $P_{\beta }$, and $P_{\text{coil}}$ represent the probabilities of the residue being part of an α-helix, β-sheet, or random coils, respectively; and $I_{\text{ASA}}$ is the binary indicator for solvent accessibility, defined as 1 when ASA≥25% and 0 otherwise.

### 2.3 Feature extraction

In this study, features derived from protein residue contact maps and ligand SMILES representations were extracted using graph-based neural networks tailored to each modality. Protein graphs were encoded using a graph attention network (GAT) with multi-head attention, which enables adaptive weighting of residue–residue interactions and better captures heterogeneous and long-range structural dependencies. In contrast, ligand graphs were modeled using a modified GraphSAGE framework, as its neighborhood aggregation mechanism effectively captures local chemical topology and supports multi-scale structural feature learning.

#### 2.3.1 Molecular feature extraction

To efficiently capture topological structures at multiple spatial scales, we developed a modified GraphSAGE framework for molecular representation learning. The original stochastic neighbor sampling was replaced with a deterministic dilated sampling strategy, in which the dilation factor increased exponentially $2^{k}$ across layers. This design enabled the model to aggregate structural information over progressively larger molecular subgraphs—directly bonded atoms in the first layer $2^{0}$, two-bond neighbors in the second layer $2^{1}$, and four-bond neighbors in the third layer $2^{2}$—thereby enhancing its ability to capture both local chemical features and long-range dependencies.

At each layer, a learnable transformation matrix *W* followed by a non-linear LeakyReLU activation function σ was applied to the mean-pooled features of the sampled neighborhood. The final output yielded a 128-dimensional atomic-level representation for each node:


(3) $$h_{\text{ligand}}^{i}=\sigma \left(W\cdot \text{MeanPool}\left(h_{\text{ligand}}^{u}|u\in N_{2^{k}}(i)\right)\right)$$

where $h_{\text{ligand}}^{i}$ denotes the 128-dimensional atomic-level representation of node *i* in the ligand; $W\in R^{128\times 128}$ is the learnable parameter weight matrix; $N_{2^{k}}$ denotes the sampled neighborhood with dilation $2^{k}$ at layer *k* with a dilation factor of $2^{k}$ (k∈{0,1,2} corresponds to local, medium-range, and long-range structural scales, respectively); and $h_{\text{ligand}}^{u}$ denotes the node feature of neighbor *u* from the previous layer.

Following convolution, a four-head attention pooling mechanism was applied to emphasize chemically important atoms. Each attention head employed an independent query vector $Q^{\left(m\right)}\in R^{1\times 128}$ to compute atomic importance weights $\beta^{\left(m\right)}$, and the results from all heads were concatenated and projected into a 512-dimensional global feature vector:


(4) $$\beta_{i}^{\left(m\right)}=\text{Softmax}\left(\frac{Q^{\left(m\right)}h_{\text{ligand}}^{i}}{\sqrt{128}}\right)$$

(5) $$h_{\text{ligand}}^{\prime }=W_{\text{pool}}\cdot \text{Concat}\left(\sum_{i}\beta_{i}^{\left(1\right)}h_{\text{ligand}}^{i},\ldots ,\sum_{i}\beta_{i}^{\left(4\right)}h_{\text{ligand}}^{i}\right)$$

where $W_{\text{pool}}\in R^{512\times 512}$ is the projection matrix and $h_{\text{ligand}}^{\prime }$ denotes the final extracted global feature of the ligand.

#### 2.3.2 Protein feature extraction

Protein (enzyme) features $h_{\text{enzyme}}$ were processed using a two-layer GAT to extract informative residue-level representations. Each GAT layer employed a multi-head attention mechanism with four parallel attention heads (K=4), which shared parameters but computed attention scores independently to enhance representational capacity.

Each attention head computed scaled dot-product attention by projecting the 24-dimensional input features $h_{\text{enzyme}}$ into 32-dimensional query and key vectors through learnable weight matrices $W_{q}^{\left(d\right)}$ and $W_{k}^{\left(d\right)}$. The attention score $e_{mn}^{\left(d\right)}$ between nodes *m* and *n* in head *k* was calculated as:


(6) $$e_{mn}^{\left(d\right)}=\frac{{\left(W_{q}^{\left(d\right)}h_{\text{enzyme}}^{m}\right)}^{T}\left(W_{k}^{\left(d\right)}h_{\text{enzyme}}^{n}\right)}{\sqrt{32}}$$

where $W_{q}^{\left(d\right)}\in R^{32\times 24}$ and $W_{k}^{\left(d\right)}\in R^{32\times 24}$ are the learnable projection matrices for the *k*-th attention head, $h_{\text{enzyme}}^{m}$ and $h_{\text{enzyme}}^{n}$ denote the 24-dimensional residue feature vectors of nodes *m* and *n*, respectively.

The attention coefficients were normalized using the Softmax activation function over all neighbors of node *m*:


(7) $$\alpha_{mn}^{\left(d\right)}={\text{Softmax}}_{n}\left(e_{mn}^{\left(d\right)}\right)$$

where $\alpha_{mn}^{\left(d\right)}$ represents the normalized attention coefficient between nodes *m* and *n* in head *d*.

The coefficients were subsequently employed to aggregate neighboring features through a learnable 32-dimensional value projection matrix $W_{v}^{\left(k\right)}$:


(8) $$h_{m}^{\left(k\right)}=\sum_{n\in N_{m}}\alpha_{mn}^{\left(d\right)}W_{v}^{\left(k\right)}h_{\text{enzyme}}^{n},\;h_{m}^{\left(k\right)}\in R^{32}$$

where $W_{v}^{\left(k\right)}\in R^{32\times 24}$ is the learnable value projection matrix for head *k*, and $N_{m}$ denotes the set of neighboring nodes of node *m*.

The outputs from all four attention heads were concatenated to form a 128-dimensional intermediate feature vector, which was then linearly transformed into a 128-dimensional output feature using a learnable projection matrix and a learnable bias vector:


(9) $$h_{\text{temp}}=W_{\text{proj}}\cdot \text{Concat}\left(h_{m}^{\left(1\right)},h_{m}^{\left(2\right)},h_{m}^{\left(3\right)},h_{m}^{\left(4\right)}\right)+b$$

where Concat(·) concatenates four 32-dimensional vectors into a 128-dimensional vector, $W_{\text{proj}}\in R^{128\times 128}$ is the learnable projection matrix, and $b\in R^{128}$ is the learnable bias vector.

To improve training stability and performance, a Graph Normalization (GraphNorm) layer was applied, followed by a residual connection:


(10) $$h_{\text{enzyme}}^{(m)\;*}=\text{GraphNorm}\left(h_{\text{temp}}+h_{\;\text{enzyme}}^{m}\right)$$

where $h_{\text{enzyme}}^{(m)\;*}\in R^{128}$ is the updated residue-level representation for node *m*.

Finally, global average pooling aggregated all residue-level features, and the resulting 128-dimensional vector was projected into a 512-dimensional protein representation:


(11) $$h_{\text{enzyme}}^{\prime }=\text{GlobalAvgPool}\left(\left\{h_{\text{enzyme}}^{(m)\;*}\right\}\right)W_{\text{final}}$$

where GlobalAvgPool(·) computes the mean over all residue features, and $W_{\text{final}}\in R^{128\times 512}$ is the final learnable projection matrix.

### 2.4 Interaction prediction

The interaction prediction module comprised two main steps: feature fusion and affinity prediction. In the fusion stage, protein and ligand representations were integrated through a bidirectional cross-attention mechanism to capture their mutual dependencies. In the prediction stage, the fused representation was passed to a lightweight classifier to estimate the protein–ligand inhibitor classification.

#### 2.4.1 Feature fusion

The 512-dimensional protein features $h_{\text{enzyme}}^{\prime }$ and molecular features $h_{\text{ligand}}^{\prime }$ were integrated using a bidirectional cross-attention mechanism, enabling the model to capture deeper enzyme–ligand interactions. In the protein-to-ligand direction, three independent linear transformations were first used to project protein features into query vectors $Q_{p}$, and ligand features into key vectors $K_{l}$ and value vectors $V_{l}$:


(12) $$Q_{p}=h_{\text{enzyme}}^{\prime }W_{q}^{p},\;K_{l}=h_{\text{ligand}}^{\prime }W_{k}^{l},\;V_{l}=h_{\text{ligand}}^{\prime }W_{v}^{l}$$

where $W_{q}^{p},W_{k}^{l},W_{v}^{l}\in R^{512\times 512}$ are the learnable projection matrices for query, key, and value, respectively.

Each of the four attention heads then computed a similarity matrix in a 128-dimensional subspace:


(13) $$E^{p2l,(m)}=\frac{Q_{p}^{\left(m\right)}{\left(K_{l}^{\left(m\right)}\right)}^{⊤}}{\sqrt{128}}$$

where $Q_{p}^{\left(m\right)},K_{l}^{\left(m\right)}\in R^{1\times 128}$ are the query and key vectors for head *m*.


(14) $$A^{p2l,(m)}=\text{Softmax}\left(E^{p2l,(m)}\right)$$

where the Softmax is over the key sequence dimension.


(15) $$C^{p2l}=\text{Concat}\left(\sum A^{p2l,(1)}V_{l}^{\left(1\right)},\ldots ,\sum A^{p2l,(4)}V_{l}^{\left(4\right)}\right)W_{o}^{p}$$

where $W_{o}^{p}\in R^{512\times 512}$ is a learnable output projection matrix.

A residual connection and layer normalization were applied to fuse the context with the original protein features:


(16) $$h_{\text{enzyme}}^{\text{out}}=\text{LayerNorm}(h_{\text{enzyme}}^{\prime }+C^{p2l})$$

In the ligand-to-protein direction, the same procedure was applied symmetrically to produce $h_{\text{ligand}}^{\text{out}}$.

The interaction between residue and atom features is first modeled at the node level using bidirectional cross-attention, producing updated node representations. Subsequently, a global mean pooling layer is applied to obtain graph-level representations for both the protein and the ligand, which are then concatenated:


(17) $${\bar{h}}_{p}=\frac{1}{N_{p}}\sum_{i=1}^{N_{p}}h_{\text{enzyme}}^{\text{out},i},\;{\bar{h}}_{l}=\frac{1}{N_{l}}\sum_{j=1}^{N_{l}}h_{\text{ligand}}^{\text{out},j}$$

where ${\bar{h}}_{p}\in R^{512}$ and ${\bar{h}}_{l}\in R^{512}$ denote the pooled protein and ligand representations, respectively; $N_{p}$ and $N_{l}$ denote the numbers of residues and atoms.

Then the joint fusion representation was defined as:


(18) $$F_{\text{fusion}}=\text{Concat}({\bar{h}}_{p},{\bar{h}}_{l})$$

where $F_{\text{fusion}}\in R^{1024}$ is the concatenated joint representation.

#### 2.4.2 Affinity prediction

In the prediction stage, the 1024-dimensional joint feature representation $F_{\text{fusion}}$ was first compressed into a 512-dimensional vector through an MLP, followed by a LeakyReLU activation function to enhance nonlinear expressiveness. Batch normalization was then applied to mitigate internal covariate shift, and dropout was used to reduce overfitting, producing the intermediate feature $h_{1}$:


(19) $$h_{1}=\text{BatchNorm}\left(\text{LeakyReLU}\left(W_{1}F_{\text{fusion}}{+b}_{1}\right)\right),$$

where $W_{1}\in R^{512\times 1024}$ and $b_{1}\in R^{512}$.

Subsequently, $h_{1}$ was mapped into a 256-dimensional latent space via another MLP employing the GELU activation function:


(20) $$h_{2}=\text{LeakyReLU}\left(W_{2}\text{Dropout}\left(h_{1},0.2\right){+b}_{2}\right),$$

where $W_{2}\in R^{256\times 512}$ and $b_{2}\in R^{256}$.

Finally, the refined feature $h_{2}$ was mapped to the binary classification space, and a Sigmoid function was applied to calculate the predicted probability *y*:


(21) $$y=\sigma \left(W_{3}h_{2}+b_{3}\right)\;W_{3}\in R^{2\times 256},\;b_{3}\in R^{2}$$

The binary cross-entropy (BCE) loss was then employed to measure the discrepancy between the predicted probability ${\hat{y}}_{i}$ and the ground-truth label $y_{i}$:


(22) $$L=-\frac{1}{N}\sum_{i=1}^{N}\left[y_{i}\;\text{log}({\hat{y}}_{i})+(1-y_{i})\;\text{log}(1-{\hat{y}}_{i})\right]$$

where $y_{i}\in \{0,1\}$ is the ground-truth label, ${\hat{y}}_{i}$ is the predicted probability for sample *i*, and *N* is the number of samples.

### 2.5 Evaluation methods

To evaluate the performance of MAISNet, six standard metrics were employed: accuracy, precision, recall, F1-score, Matthews correlation coefficient (MCC), and the area under the receiver operating characteristic curve (AUC). Their formal definitions are as follows:


(23) $$\text{Accuracy}=\frac{\text{TP}+\text{TN}}{\text{TP}+\text{TN}+\text{FP}+\text{FN}}$$

(24) $$\text{Precision}=\frac{\text{TP}}{\text{TP}+\text{FP}}$$

(25) $$\text{Recall}=\frac{\text{TP}}{\text{TP}+\text{FN}}$$

(26) $$F1-\text{score}=2\times \frac{\text{Precision}\times \text{Recall}}{\;\text{Precision}+\text{Recall}}$$

(27) $$\text{MCC}=\frac{\text{TP}\times \text{TN}-\text{FP}\times \text{FN}}{\sqrt{\left(\text{TP}+\text{FP})(\text{TP}+\text{FN})(\text{TN}+\text{FP})(\text{TN}+\text{FN}\right)}}$$

(28) $$\text{AUC}=\int_{0}^{1}\text{TPR}({\text{FPR}}^{-1}(x))dx$$

where TP (true positive), TN (true negative), FP (false positive), and FN (false negative) denote the number of correctly predicted inhibitors, correctly predicted non-inhibitors, incorrectly predicted inhibitors, and incorrectly predicted non-inhibitors, respectively.

To further validate model performance, we compared MAISNet with five baseline models:

- **DeepDock:** A CNN-based model using voxelized protein–ligand complexes and spatial attention to capture 3D interaction patterns (Méndez-Lucio *et al*. 2021).
- **IGN:** A graph-based framework encoding proteins and ligands as graphs, using interatomic distances and GCN layers for local and global interaction modeling (Stärk *et al*. 2022).
- **MolTrans:** A Transformer-based model that uses self- and cross-attention for molecular sequence encoding and interaction prediction (Chen *et al*. 2021).
- **HaMM**: A model with atomic- and molecular-level attention modules, using bidirectional cross-attention to optimize feature interactions for improved prediction (Li *et al*. 2023a).
- **3D-CNN:** A convolutional model processing voxelized 3D structures to capture complex spatial and structural patterns in protein–ligand interactions (Ji *et al*. 2013).

To ensure fair comparison, baseline models were implemented according to their original architectures and hyperparameters.

## 3 Experiments and results

### 3.1 Experimental settings

All experiments were conducted in Python 3.10 using the PyTorch 2.1.0 framework and executed on an Ubuntu 22.04 operating system. The hardware environment consisted of an NVIDIA RTX 4090 GPU with 24 GB of VRAM and an AMD EPYC 9654 96-core CPU running at 2.40 GHz.

For all models, the training was conducted for 100 epochs with a batch size of 32. Dropout (0.2) was applied to the output layers of both the GAT and GraphSAGE modules. The Adam optimizer was used with a learning rate of 0.001 and weight decay of 1 e−5. A linear warm-up strategy was applied during the first 10 epochs, increasing the learning rate from 10% to its initial value, followed by a ReduceLROnPlateau scheduler that reduced the learning rate by a factor of 0.7 after five consecutive epochs with no improvement.

### 3.2 Performance evaluation

**Figure 3:**
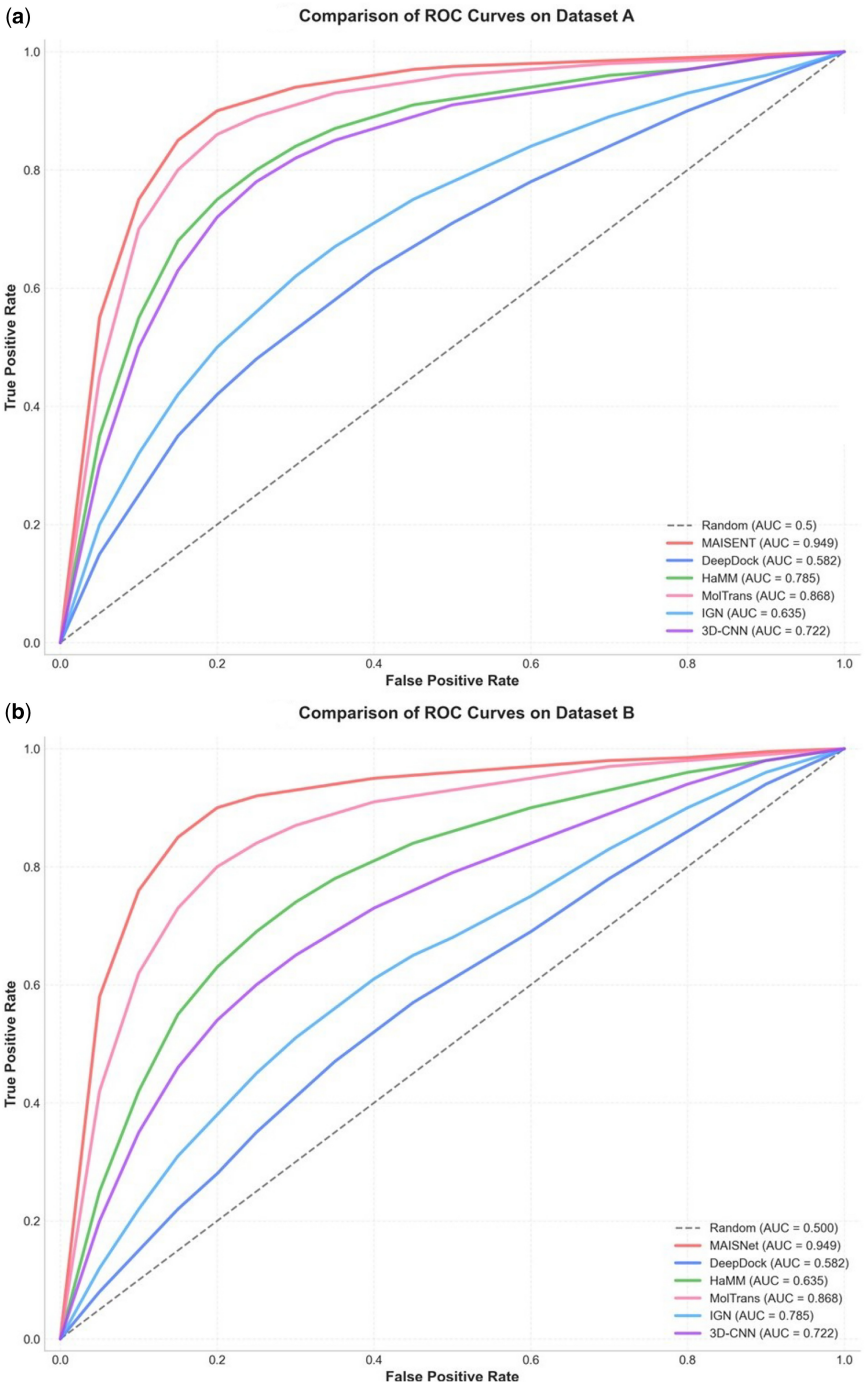
Performance comparison of the existing models. (a) ROC curve comparison of six methods on the testing set of Dataset A. (b) ROC curve comparison of six methods on Dataset B.

We compared MAISNet with five state-of-the-art methods—DeepDock, IGN, MolTrans, HaMM, and 3D-CNN—on the testing set of Dataset A and Dataset B, respectively. The same data splits and parameter settings as reported in the original studies were adopted. Model performance was assessed using six metrics: accuracy, precision, recall, F1-score, MCC, and AUC, as shown in Table 2. The ROC curves depicting model performance are shown in Fig. 3.

**Table 2:** Performance comparison of different methods across Dataset A and Dataset B after medicinal chemistry filtering.

Methods	Accuracy (95% CI)	Precision (95% CI)	Recall (95% CI)	F1-score (95% CI)	MCC (95% CI)	AUC (95% CI)
Dataset A (*N* = 17 352)						
DeepDock	0.732 (0.725–0.739)	0.735 (0.728–0.742)	0.728 (0.721–0.735)	0.731 (0.724–0.738)	0.465 (0.457–0.473)	0.725 (0.718–0.732)
HaMM	0.815 (0.809–0.821)	0.810 (0.804–0.816)	0.805 (0.799–0.811)	0.807 (0.801–0.813)	0.631 (0.624–0.638)	0.895 (0.890–0.900)
MolTrans	0.902 (0.898–0.906)	0.895 (0.890–0.900)	0.908 (0.904–0.912)	0.901 (0.897–0.905)	0.805 (0.799–0.811)	0.912 (0.908–0.916)
IGN	0.685 (0.678–0.692)	0.702 (0.695–0.709)	0.640 (0.633–0.647)	0.670 (0.663–0.677)	0.375 (0.367–0.383)	0.782 (0.776–0.788)
3D-CNN	0.702 (0.695–0.709)	0.715 (0.708–0.722)	0.865 (0.860–0.870)	0.783 (0.777–0.789)	0.442 (0.434–0.450)	0.665 (0.658–0.672)
MAISNet	0.924 (0.920–0.928)	0.918 (0.914–0.922)	0.935 (0.931–0.939)	0.926 (0.922–0.930)	0.849 (0.844–0.854)	0.951 (0.948–0.954)
Dataset B (*N* = 5838)						
DeepDock	0.641 (0.629–0.653)	0.612 (0.600–0.624)	0.675 (0.663–0.687)	0.642 (0.630–0.654)	0.285 (0.273–0.297)	0.582 (0.569–0.595)
HaMM	0.662 (0.650–0.674)	0.685 (0.673–0.697)	0.745 (0.734–0.756)	0.714 (0.702–0.726)	0.332 (0.320–0.344)	0.785 (0.774–0.796)
MolTrans	0.845 (0.836–0.854)	0.812 (0.802–0.822)	0.840 (0.831–0.849)	0.826 (0.816–0.836)	0.695 (0.684–0.706)	0.868 (0.859–0.877)
IGN	0.552 (0.539–0.565)	0.595 (0.582–0.608)	0.510 (0.497–0.523)	0.550 (0.537–0.563)	0.115 (0.103–0.127)	0.635 (0.623–0.647)
3D-CNN	0.710 (0.698–0.722)	0.692 (0.680–0.704)	0.732 (0.721–0.743)	0.711 (0.699–0.723)	0.421 (0.409–0.433)	0.722 (0.710–0.734)
MAISNet	0.906 (0.898–0.914)	0.905 (0.897–0.913)	0.912 (0.905–0.919)	0.908 (0.901–0.915)	0.822 (0.812–0.832)	0.949 (0.943–0.955)

Abbreviations: CI, confidence interval; AUC, area under the ROC curve; MCC, Matthews correlation coefficient.

On the testing set of Dataset A, MAISNet achieved the best overall performance, with an accuracy of 0.924 (95% CI: 0.920–0.928), precision of 0.918 (95% CI: 0.914–0.922), recall of 0.935 (95% CI: 0.931–0.939), F1-score of 0.926 (95% CI: 0.922–0.930), MCC of 0.849 (95% CI: 0.844–0.854), and AUC of 0.951 (95% CI: 0.948–0.954). Compared with the strongest baseline (MolTrans), MAISNet improved accuracy by 2.2%, F1-score by 2.5%, and MCC by 4.4%. Notably, compared with IGN, which performed worst in Recall (0.640), MAISNet achieved a substantial 29.5% absolute improvement.

On Dataset B, MAISNet continued to maintain strong generalization, reaching an accuracy of 0.906 (95% CI: 0.898–0.914), precision of 0.905 (95% CI: 0.897–0.913), recall of 0.912 (95% CI: 0.905–0.919), F1-score of 0.908 (95% CI: 0.901–0.915), MCC of 0.822 (95% CI: 0.812–0.832), and AUC of 0.949 (95% CI: 0.943–0.955). These results consistently surpassed those of all baseline models, underscoring MAISNet’s robustness and adaptability to varying data distributions beyond the initial training domain.

### 3.3 Ablation experiments

To evaluate the contribution of each key component in MAISNet, ablation experiments were conducted on Dataset A (testing set) and Dataset B using six evaluation metrics (accuracy, precision, recall, F1-score, MCC, and AUC). Specifically, we removed the bidirectional cross-attention module (No_attention) and replaced the multi-scale graph convolution module with a single-scale GNN (SingleGNN), with results summarized in Table 3.

**Table 3:** Performance comparison of MAISNet and its ablated variants across Dataset A and Dataset B.

Methods	Accuracy (95% CI)	Precision (95% CI)	Recall (95% CI)	F1-score (95% CI)	MCC (95% CI)	AUC (95% CI)
Dataset A (*N* = 17 352)						
No_attention	0.810 (0.804–0.816)	0.811 (0.805–0.817)	0.851 (0.846–0.856)	0.830 (0.824–0.836)	0.620 (0.613–0.627)	0.896 (0.891–0.901)
SingleGNN	0.780 (0.774–0.786)	0.839 (0.834–0.844)	0.742 (0.735–0.749)	0.788 (0.782–0.794)	0.575 (0.568–0.582)	0.863 (0.858–0.868)
MAISNet	0.924 (0.920–0.928)	0.918 (0.914–0.922)	0.935 (0.931–0.939)	0.926 (0.922–0.930)	0.849 (0.844–0.854)	0.951 (0.948–0.954)
Dataset B (*N* = 5838)						
No_attention	0.780 (0.769–0.791)	0.775 (0.764–0.786)	0.801 (0.791–0.811)	0.790 (0.780–0.800)	0.570 (0.557–0.583)	0.831 (0.821–0.841)
SingleGNN	0.741 (0.730–0.752)	0.802 (0.792–0.812)	0.719 (0.707–0.731)	0.750 (0.739–0.761)	0.530 (0.517–0.543)	0.797 (0.787–0.807)
MAISNet	0.906 (0.898–0.914)	0.905 (0.897–0.913)	0.912 (0.905–0.919)	0.908 (0.901–0.915)	0.822 (0.812–0.832)	0.949 (0.943–0.955)

Abbreviations: CI, confidence interval; AUC, area under the ROC curve; MCC, Matthews correlation coefficient.

The ablation results highlight the critical contribution of both the bidirectional cross-attention and multi-scale GNN modules. Removing the attention mechanism (No_attention) led to a significant performance decline, with accuracy dropping by 11.4% on Dataset A and 12.6% on Dataset B, while MCC decreased by 22.9% and 25.2%, respectively. This confirms that cross-attention is essential for capturing fine-grained protein–ligand interactions. Furthermore, utilizing a single-scale GNN (SingleGNN) resulted in even steeper reductions, with accuracy falling by 14.4% on Dataset A and 16.5% on Dataset B. These findings underscore that multi-scale feature extraction is vital for capturing the diverse molecular patterns necessary for accurate AChEIs classification.

To further evaluate the effectiveness of the multi-species integration strategy, we conducted an additional ablation experiment by training a single-species model using only *Homo sapiens* AChE data (denoted as Single-species) and comparing it with the original multi-species model (Multi-species) under identical datasets and evaluation metrics (Table 4).

**Table 4:** Performance comparison of multi-species MAISNet and its single-species ablated variant across Dataset A and Dataset B.

Methods	Accuracy (95% CI)	Precision (95% CI)	Recall (95% CI)	F1-score (95% CI)	MCC (95% CI)	AUC (95% CI)
Dataset A (*N* = 17 352)						
Multi_species	0.924 (0.920–0.928)	0.918 (0.914–0.922)	0.935 (0.931–0.939)	0.926 (0.922–0.930)	0.849 (0.844–0.854)	0.951 (0.948–0.954)
Single_species	0.700 (0.693–0.707)	0.763 (0.757–0.769)	0.731 (0.724–0.738)	0.729 (0.722–0.736)	0.600 (0.593–0.607)	0.788 (0.782–0.794)
Dataset B (*N* = 5838)						
Multi_species	0.906 (0.898–0.914)	0.905 (0.897–0.913)	0.912 (0.905–0.919)	0.908 (0.901–0.915)	0.822 (0.812–0.832)	0.949 (0.943–0.955)
Single_species	0.753 (0.742–0.764)	0.722 (0.710–0.734)	0.758 (0.747–0.769)	0.747 (0.736–0.758)	0.559 (0.546–0.572)	0.800 (0.790–0.810)

Abbreviations: CI, confidence interval; AUC, area under the ROC curve; MCC, Matthews correlation coefficient.

The results confirm the critical role of multi-species integration in MAISNet. Compared with the Single_species variant (trained only on *Homo sapiens*), the multi-species model achieved markedly higher performance, with accuracy and AUC decreasing by 22.4% and 16.3% on Dataset A, and by 15.3% and 14.9% on the external Dataset B when trained using single-species data. These findings indicate that cross-species data integration alleviates data scarcity and significantly improves model generalization.

### 3.4 Inhibitor screening and molecular docking

To validate the practical applicability of MAISNet in identifying potent AChEIs, we conducted a screening workflow that combined MAISNet-based predictions with structure-based molecular docking.

### 3.5 Molecular and protein structure preparation

A total of 51 220 unvalidated small molecules were retrieved from the ZINC database (https://zinc.docking.org/, accessed 1 July 2025) (Irwin and Shoichet 2005) for inhibitor screening. Molecule preprocessing followed the same criteria as Dataset A, including SMILES string standardization and retention of molecules with at least one active pharmacophore.

For the target protein, human AChE (UniProt ID: P22303) was chosen due to its relevance in neurodegenerative diseases. The 3D structure was retrieved from the AlphaFold2 database (https://alphafold.ebi.ac.uk/, accessed 28 June 2025) (Jumper *et al*. 2021). For proteins with over 300 residues, we focused on regions around the active pocket to preserve structural integrity and highlight key functional areas.

### 3.6 MAISNet-based candidate screening and docking

Each molecule was assigned a predicted confidence score representing the likelihood of being a potent AChE inhibitor. To ensure high reliability, a confidence threshold of 0.97 was applied. This screening identified 405 candidate molecules that passed the criteria for potential inhibitors. These molecules were selected based on the same medicinal chemistry filters used during dataset curation, which prioritize drug-like physicochemical properties and essential pharmacophoric features for AChE binding, resulting in a final set of 321 potential inhibitors.

Subsequent virtual docking was performed using the CB-Dock2 platform (https://cadd.labshare.cn/cb-dock2/php/index.php, accessed 13 August 2025) (Liu *et al*. 2022), with the docking grid centered on AChE’s active pocket. Binding affinities were assessed using Vina scores, and molecules with scores below −9 kcal/mol were considered high-affinity candidates. The top two molecules, exhibiting the strongest binding to human AChE, are summarized in Table 5.

**Table 5:** Molecular docking results and 2D structures of top-ranked compounds.

Rank	SMILES	Vina score	Predicted	2D structure
		(kcal/mol)	Probability	
1	COC(=O)c1c(C)[nH]c(C(=O)[C@@H] (C)NCC23CC4CC(CC(C4)C2)C3)c1C .	−10.1	.980	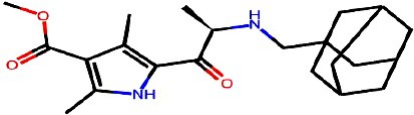
2	COP(=O)(CCN1C(=O)NC(=O)[C@@H]2 [C@@H]1N=CN2C)OC .	−9.9	.973	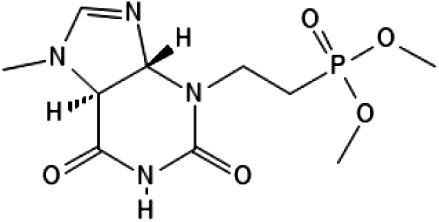

**Figure 4:**
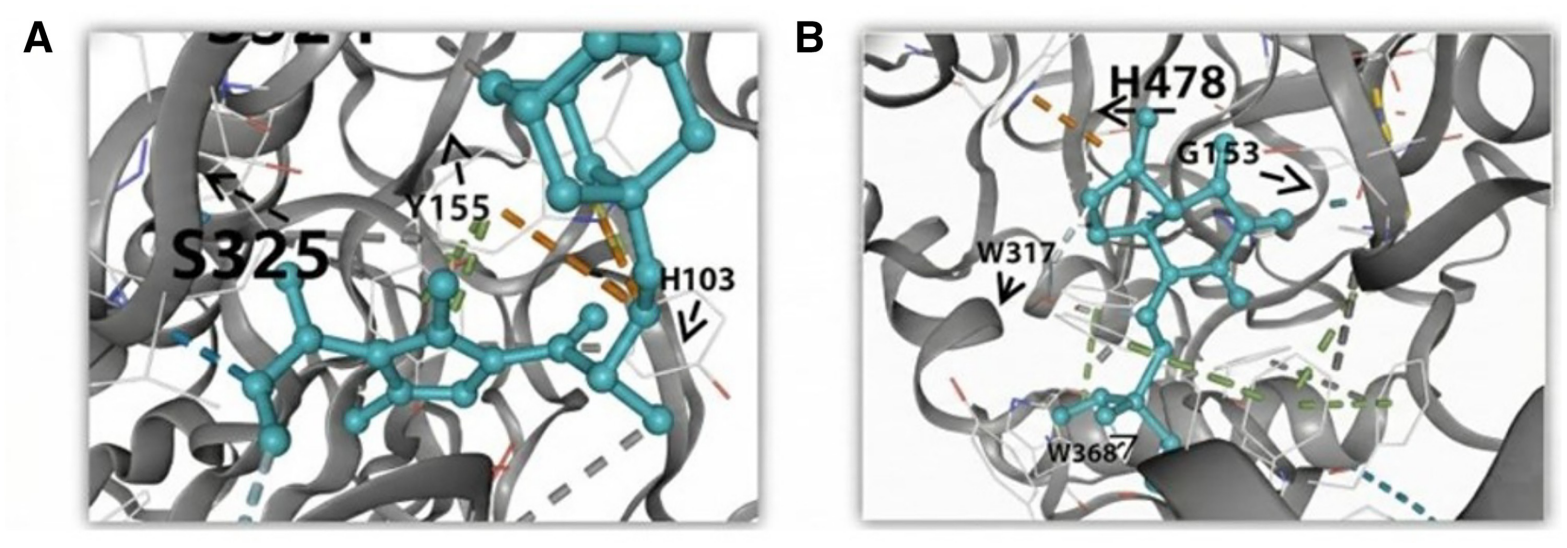
Binding poses of the top two candidate molecules within the catalytic pocket of human AChE (P22303). (a) Methyl 2-[(3S)-3–(1, 2, 3, 4, 5, 6, 7, 8-octahydro-2-naphthyl)-2-(methoxycarbonyl)-1H-pyrrol-1-yl]acetate. (b) Dimethyl (2–5-methyl-4,6-dioxo-1,3a, 4,6a-tetrahydroimidazo [4,5-d] imidazol-5-yl ethyl) phosphonate.

Figure 4 shows the binding poses of the top two candidate molecules in the catalytic pocket of human AChE. Key interactions, such as hydrogen bonds (e.g. residues F328, S324) and hydrophobic contacts, stabilize the ligand and contribute to high inhibitor classification. These docking results validate MAISNet’s predictions, confirming that the molecules occupy critical AChE regions and form favorable interactions. Among them, the Methyl 2-[(3S)-3–(1, 2, 3, 4, 5, 6, 7, 8-octahydro-2-naphthyl)-2-(methoxycarbonyl)-1H-pyrrol-1-yl]acetate, with the highest predicted confidence score and strongest binding affinity, was identified as the representative AChE inhibitor.

## 4 Discussion

In this study, we presented MAISNet, a deep learning framework specifically developed for screening AChE inhibitors. By utilizing a multi-species approach, MAISNet enhanced the model’s generalization ability. The incorporation of structural features allowed the model to better capture the spatial and stereochemical properties of protein–ligand interactions, leading to more accurate and robust predictions. Compared with state-of-the-art methods, MAISNet demonstrated superior robustness, generalization ability, and predictive accuracy.

MAISNet successfully predicted two high-affinity AChE inhibitors based on their high-predicted confidence scores and strong binding affinities, which were further validated through molecular docking. This highlights the practical value of MAISNet in drug discovery.

Nevertheless, several limitations remain. Firstly, although MAISNet demonstrated robust *in silico* performance, its generalizability requires validation on larger and more diverse datasets. Secondly, the current findings are based solely on computational predictions and lack *in vitro* verification. Thirdly, the model does not account for the temporal dynamics of protein–ligand interactions, which may affect inhibitor efficacy under physiological conditions. Future studies should aim to integrate experimental validation, incorporate dynamic binding features, and further refine the model architecture to enhance interpretability and translational applicability.

## 5 Conclusions

In summary, MAISNet introduced an innovative computational framework that accelerated the discovery of AChE inhibitors. By integrating GAT, GraphSAGE, and a bidirectional cross-attention mechanism, the model achieved precise and robust representations of protein–ligand interactions. Its multi-species learning strategy and residue contact map representations enhanced cross-dataset transferability, enabling accurate and generalizable screening performance. Compared with existing approaches, MAISNet consistently outperformed baseline methods across multiple evaluation metrics and successfully identified two high-affinity inhibitors targeting human AChE.

Looking forward, further optimization of the model architecture and training strategies will expand its applicability to broader classes of bioactive molecules. Incorporating experimental validation data will not only strengthen predictive reliability but also provide biological interpretability, thereby bridging computational predictions with translational applications. Overall, MAISNet offers an efficient and reliable platform for accelerating drug discovery, contributing valuable insights to therapeutic development for neurodegenerative diseases.

## Data Availability

Code that supports the reported results can be found at: https://github.com/liangshengjie111/MAISNet. The archival version of the code is preserved on Zenodo at https://doi.org/10.5281/zenodo.18721665.
